# Completing the Picture: Importance of Considering Participatory Mapping for REDD+ Measurement, Reporting and Verification (MRV)

**DOI:** 10.1371/journal.pone.0166592

**Published:** 2016-12-15

**Authors:** Guillaume Beaudoin, Serge Rafanoharana, Manuel Boissière, Arief Wijaya, Wahyu Wardhana

**Affiliations:** 1 Center for International Forestry Research (CIFOR), Jl. CIFOR, Situ Gede, Bogor, Indonesia; 2 Centre de coopération Internationale en Recherche Agronomique pour le Développement (CIRAD), Campus de Baillarguet Montpelier Cedex 5, France; 3 Faculty of Forestry GadjahMada University (UGM), Jl. Agro Bulaksumur, n°1 Sleman-Yogyakarta, Indonesia; Pacific Northwest National Laboratory, UNITED STATES

## Abstract

Remote sensing has been widely used for mapping land cover and is considered key to monitoring changes in forest areas in the REDD+ Measurement, Reporting and Verification (MRV) system. But Remote Sensing as a desk study cannot capture the whole picture; it also requires ground checking. Therefore, complementing remote sensing analysis using participatory mapping can help provide information for an initial forest cover assessment, gain better understanding of how local land use might affect changes, and provide a way to engage local communities in REDD+. Our study looked at the potential of participatory mapping in providing complementary information for remotely sensed maps. The research sites were located in different ecological and socio-economic contexts in the provinces of Papua, West Kalimantan and Central Java, Indonesia. Twenty-one maps of land cover and land use were drawn with local community participation during focus group discussions in seven villages. These maps, covering a total of 270,000ha, were used to add information to maps developed using remote sensing, adding 39 land covers to the eight from our initial desk assessment. They also provided additional information on drivers of land use and land cover change, resource areas, territory claims and land status, which we were able to correlate to understand changes in forest cover. Incorporating participatory mapping in the REDD+ MRV protocol would help with initial remotely sensed land classifications, stratify an area for ground checks and measurement plots, and add other valuable social data not visible at the RS scale. Ultimately, it would provide a forum for local communities to discuss REDD+ activities and develop a better understanding of REDD+.

## Introduction

Recent progress in mapping forest carbon biomass, using remote sensing, has been considered important and broadly applied. For example, Asner et al. [1] mapped the above ground carbon density for the whole of Peru. Other current research has even quantified global forest change [2]. With such advancement in science, Remote Sensing (RS) and Geographical Information System (GIS) tools have been widely promoted in REDD+ Measurement, Reporting and Verification (MRV) to estimate the baseline for carbon emissions and to monitor forest change [3,4], in combination with on-the-ground methods [3,5].

But something is missing from these maps. A forest is not only trees and carbon biomass; it is also people living in it and from it. Worldwide, millions of people depend on the forest for their livelihoods, food, timber and land expansion for agriculture [6]. It is therefore important that MRV capture the activities of local people as it may well play a major role in shaping forest cover.

While remote sensing analysis provides a quick and precise assessment of the forest cover on a large scale, it has more difficulty capturing locally driven changes and small-scale deforestation that might cause significant changes in land cover (LC). So, how can local information be incorporated into RS maps? Rindfuss and Stern [7] suggested a combination of social science and remote sensing approaches to provide a more complete picture of the situation on the ground.

Participatory Mapping (PM), a process of using local perceptions and knowledge to build maps of a shared geographical location, can be used to provide a social context to remote sensing analysis. The following examples are a brief representation of the many forms and uses of participatory maps: Balram et al. [8] used collaborative GIS methods for integrating local knowledge to establish biodiversity conservation priorities; Sheil et al. [9] used community mapping to gather information on natural resources, special sites (e.g. sacred sites) and local perceptions of a shared geographical framework; Hossain et al [10] integrated RS, GIS and PM to zone coastal resource use in Bangladesh; and Mapedza et al. [11] used participatory maps to investigate Land Cover Change (LCC) and to capture human activities that lead to LCC in Zimbabwe.

Using PM, we wanted to offer evidence that local communities’ extensive knowledge of the landscape can provide a social context for MRV and help complete RS initial land/forest cover assessments, essential if we are to understand the local drivers of forest change.

The research for this paper was part of a larger study looking at the conditions under which participation in REDD+ MRV (PMRV) is feasible and sustainable [12]. This study took place in Indonesia, which has progressed in its REDD+ readiness, especially regarding policy, legal and institutional frameworks [13]. The creation of the Indonesian National REDD+ Agency in September 2013 [14] seemed a promising step forward. However, Indonesia is still behind in its REDD+ MRV readiness compared to other countries, like Peru and Brazil, especially in terms of remote sensing (RS) and GIS capacity [4], which are predominantly based on desk studies. Furthermore, merging the REDD+ Agency with the Ministry of Environment and Forestry in 2015 [15] could be considered a step backwards in the implementation of the REDD+ agenda.

Community participation in REDD+ is another aspect of this ‘readiness’ lagging behind even though the United Nations Framework Convention on Climate Change (UNFCCC) stipulated that indigenous people and local communities should be fully engaged in effective participation in REDD+ [16]. Recently, Joseph et al. [4] highlighted the need to maximize community participation in MRV, while others suggest it will improve social safeguards [17], aid benefit sharing [18, 19] and reduce monitoring costs [20, 21]. With all these considerations, involving local communities in mapping, or other carbon estimation activities, could lead to more effective, long-term community participation in REDD+ MRV

In this article we illustrate what information can be gained from local participation and how much participatory mapping can add to forest mapping. While Vergera-Asenjo et al. 2014 [22], demonstrated that indigenous people could add to the accuracy of land cover identification, we focused on the content of local contributions rather than accuracy.

- We illustrate how participatory mapping can complement initial remote sensing land cover classification and assist ground checks and future RS mapping.
- We show how correlations between Land Use (LU) and land cover, obtained from participatory maps and remote sensing data, are used to interpret changes in forest cover.
- We also use historical context to explain these changes, which can be helpful for time series analysis.

We provide examples and present our results for three Indonesian provinces (Papua, West Kalimantan and Central Java) to illustrate the individuality of each landscape where local communities still depend on the forest for their livelihoods and could therefore benefit from participating in REDD+. It should be noted that additional technical and traditional knowledge was only made available through local participation.

## Research sites

**Fig 1:**
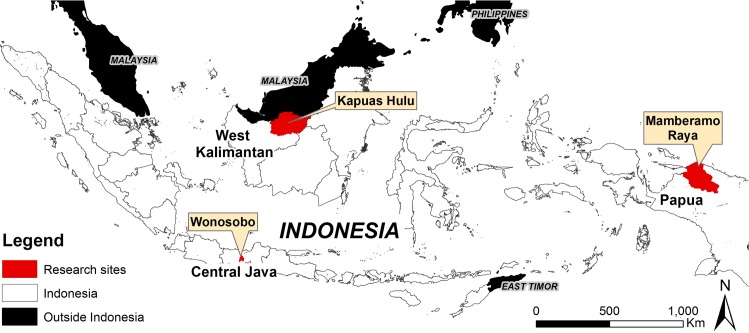
Map of research sites in Indonesia

This study was conducted in three districts: Mamberamo-Raya, Papua Province; Kapuas Hulu, West Kalimantan Province; and Wonosobo, Central Java Province, Indonesia (Fig 1).

These three provinces are at different stages of the forest transition curve (Table 1) as defined by Mather [23]. Papua, with the most important natural forest, has the smallest population, lowest accessibility and high economic pressure. West Kalimantan is in the middle of the transition moving towards more planted forests and higher deforestation, with an important secondary forest area. It has better access, higher population density and medium economic pressure. Central Java is on the ‘recovery’ side as natural forest has all but disappeared, but villagers are replanting forests. It is among the most densely populated and economically developed provinces in Indonesia.

**Table 1:** Research site description of forest and socio-economic conditions

Site description		PAPUA	WEST KALIMANTAN	CENTRAL JAVA
Forest conditions*	**Forested area kha**	31,530	14,699	3,498
	**Remaining area of forest. kha*(%)***	Natural forest: 19,805*(63%)*; Secondary forest: 5,153 *(16%)*; Planted forest: 2*(0*.*01%)*; Non-forest: 6,868*(21%)*	Natural forest: 2,544 *(17%)*; Secondary forest: 4,064 *(28%)*; Planted forest: 12 *(0*.*1%)*; Non-forest: 7,952 *(55%)*	Natural forest: 0 (0.003%); Secondary forest: 84 (3%); Planted forest: 1,034 (30%); Non-forest: 2,341 (67%)
	**Deforestation between 2009–2011 ha/Year *(%)***	Natural forest: 6,800*(0*.*02%)*; Secondary forest: 3,577*(0*.*01%)*	Natural forest: 586 *(0*.*004%)*; Secondary forest: 41,152*(0*.*30%)*	Secondary forest: 499 (0.01%); Planted forest: 3,739 (0.11%)
	**Dominant forest regime kha*(%)***	Conservation: 7,755 *(25%)*; Protected 7,815 *(25%)*; Production: 14,817*(45%)*; Others: 2,162 *(5%)*	Conservation: 1,646*(11%)*; Protected: 2,307 *(16%)*; Production: 5,226 *(35%)*; Others: 5,708 *(38%)*	Conservation: 126 (3%); Protected: 84(2%); Production: 547 (15%); Others: 2,851 (80%)
Socio-economic conditions*	**Population**	Low	Medium	High
	**Accessibility**	Low	Medium	High
	**Economic pressure**	High	Medium	Medium

*Note: Forest conditions are based on data from the Indonesian Ministry of Forestry [24], while the socio-economic conditions are based on data from the Indonesian Central Statistics Agency [25].

Table 2 provides information regarding the local communities who participated in the mapping activities in each province. Although there were important variations in ethnic groups and dependency on natural resources, we noticed that most of the local people were farmers and/or hunter-gatherer, with only a few fishermen. Literacy was high in Central Java and West Kalimantan, but low in Papua. Access to the villages also varied: in Central Java, communication was well developed, with roads and telecommunication networks reaching most villages, while in West Kalimantan, the villages were often located far from the main road and phone communication was only available in certain spots of the villages. In Papua there were no roads to the villages or a telecommunication network. River transport was the only way to reach the nearest town, often taking more than a day. Despite these differences, all the participants in the mapping exercises could use the base maps, recognize the different parts of their territories and fill the gaps with their knowledge and perceptions without any problem. Most of the participants involved in the mapping exercises had received a junior high school or higher education and were also officials or had customary positions in the village such as village secretary, together with villagers that had extensive knowledge of their territory such as hunters or fishermen.

**Table 2:** Research site description of local communities status and conditions

Site	PAPUA	WEST KALIMANTAN	CENTRAL JAVA
**Village's ethnic groups**	Bagusa and Yoke	Dayak Melayu	Javanese
**Main local source of livelihoods**	Hunting (crocodiles, birds, small mammals), NTFP gathering, fishing, gardening (sago plantation), home gardening	Gardening (upland rice and vegetables), smallholder plantations (rubber), wood and NTFP extraction, small-scale hunting	Smallholder plantations (Albizia, and others), livestock, small scale NTFP gathering and hunting
**Education**	5 to 40% had low or no education, 15 to 45% had completed high school and 25–45% had received senior high school or higher education	30 to 45% had low or no education, 26 to 45% had completed junior high school and 25% had received senior high school or higher education	20% had low or no education, 60% had completed junior high school and 20% had received senior high school or higher education
**Occupation**	Village officials (e.g. secretary), customary leader (e.g. clan leader, family elder) farmers, hunters and fishermen	Village officials (head of neighbourhood RT/RW), delegates from village associations farmers, hunters, fishermen	Village officials (head of neighbourhood RT/RW) Perhutani* representatives, farmers, hunters *Indonesian state owned timber company

## Materials and Methods

This study was conducted in 7 villages (Table 3) by a multidisciplinary team comprising researchers from three disciplines: 1) social science, 2) governance and 3) RS and GIS. To conduct the fieldwork, the foreign researchers received authorisation from the Indonesian Ministry of Research and Technology (Kementerian Riset dan Teknologi, Kemenristek) and the Indonesian researchers obtained their authorisation from the Directorate General of National Unity and Politics (Kesatuan Bangsa dan Politik, Kesbangpol) of the Indonesian Ministry of Home Affairs (Kementerian Dalam Negeri, Kemendagri)

**Table 3:** Materials used to develop the base maps.

PROVINCES	PAPUA	WEST KALIMANTAN	CENTRAL JAVA
*(Villages)*	*(Bagusa and Yoke)*	*(Hulu Pengkadan*, *Nanga Jemah and Sri Wangi)*	*(Lebak and Karang Anyar)*
**BASE MAPS**			
**Features on the maps**	* Main rivers and settlements according to BPS 2011	Rivers, roads, settlements and administrative boundaries according to BPS 2011	Main rivers, water bodies, roads and settlements from World Imagery base map from ArcGIS online**. Administrative boundaries according to BPS 2011
**Scale**	1:50,000	1:10,000 and 1:30,000	1:4,000 and 1:6,000
**Satellite Image**			
**Source**	Landsat 7 (path 103 row 61)	Spot 5 and Landsat 8 (path 119 row 60)	World Imagery**
**Year**	2000	2011/2013	2008
**Resolution**	30m	10m / 30m	1m
**Scale**	1:50,000	1:10,000; 1:30,000	1:4,000
**Size of the area (ha)**	Bagusa: 90,000; Yoke: 140,000	HuluPengkadan: 2,700; Nanga Jemah: 32,400; Sri Wangi: 3,600	Lebak: 600; Karanganyar: 700

**Note**:

*Base maps for Papua were developed during previous CIFOR research [26].

**Source for World Imagery: Esri, DigitalGlobe, GeoEye, Earthstar Geographics, CNES/Airbus DS, USDA, USGS, AEX, Getmapping, Aerogrid, IGN, IGP, swisstopo, and the GIS User Community.

We describe the methods used by both the RS/GIS and social science teams to create maps using community participation and remote sensing, to interpret the overlaps and correspondence, and to correlate land use with land cover.

### Base maps

Base maps were developed from different sources of satellite images depending on the best resolution available and lowest cloud coverage. In Papua, we used Landsat 7 for year 2000 because this is the only data that provides cloud free coverage of the study area and is sufficient to generate land cover classifications. Cloud free data after 2000 were not available for these specific locations. Comparing the year 2000 images with Landsat 8 data for year 2013, which had more than 65% cloud cover, we found no dramatic changes during this period.

Ancillary data such as main rivers, settlements and boundaries were obtained from the Indonesian Central Statistics Agency [25] (Table 3). Village boundaries for each village were often adjusted during the first community meeting. Throughout these meetings, positions and local names of important places and topographic elements were added to the maps and geo-referenced.

### Participatory mapping

Prior to our fieldwork, all team members agreed on common ethical guidelines: authorisation must be sought not only from the government of Indonesia at the different governance levels, but also from village communities including village authorities. Once we arrived in each village (each visit), we reported to the village government, customary and religious authorities. We introduced our research, explained how the research outcomes would be used and asked permission to conduct the research. This information and our request were then presented during community meetings. Six villages granted permission verbally, while the remaining one asked for a written statement promising not to use the maps for anything other than our research. These villages also requested an explanation of concepts such as carbon and climate change before allowing us to start our activities.

Once permission to conduct the research was obtained we conducted a series of four (Land Cover (LC) mapping, Land Use (LU) mapping, historical LU/LC and finalization) Focus Group Discussions (FGD) in each village with 6 to 12 participants (men and women, of different ages, in one group). Each FGD was assigned a facilitator (researcher) who helped with drawing when necessary. We stated that participation was voluntary and open to all villagers. Consequently, village men and women, young and old who were heads of clans, neighbourhood heads, village secretaries, farmers, midwives, fishermen and hunters participated in the FGDs. They had a wide understanding and knowledge of their territory, which they were keen to share. During the first FGD (LC), we overlaid the base maps with satellite images as described in Table 3. The base maps helped the local people to identify the different features and to delineate LC types and boundaries on the map. For each LC type the FGD participants were able to describe the dominant vegetation, using local name(s), and often the Indonesian vernacular name(s). Once the LC map was completed, a second FGD (with the same respondents) for the LU map was conducted during which information on each land use was collected (e.g. what kind of crops are planted in the gardens, do they hunt in all LC types).

The next step was a field check. Using the Global Positioning System (GPS), together with two or more villagers who had attended the FGD, main topographical features (rivers, hills), infrastructure (roads, settlements) and LU/LC types were geo-located. In total, 534 GPS points were collected from all sites: 34 GPS points in Papua (participatory maps had already been made and geo-referenced from a previous project: COLUP [26]); 101 for Central-Java and 399 GPS points in West-Kalimantan. Once the present LC and LU maps were completed, maps describing historical LU/LC were generated using FGDs.

The final step of the participatory mapping process was to clean the draft maps and ask for final amendments from villagers during a final FGD. For this purpose we displayed the temporary maps in public places for all villagers to review, give their comments and check the validity of the maps. We also asked for authorization to use these maps for our research purposes during a community meeting. We visited each village twice during 2013 and the digitized maps were given to the villagers during the second visit.

### Remote sensing analysis

For our study, remote sensing analysis was conducted entirely as a desk study in order to emulate how Indonesian government agencies conduct RS for REDD+ MRV.

As the satellite images covered larger areas than the study location we had to reduce the size of the image to cover only the area of interest. For this we used a vector file, which defines the boundaries, was used to subset all satellite images. To produce the RS maps, a pre-processing of Landsat and SPOT data was conducted to reduce atmospheric disturbances. To produce cloud free coverage data, we filtered haze, clouds and shadows using ERDAS IMAGINE®2011 software (Leica Geo systems, Atlanta, Georgia, USA). The widely used dark object subtraction (DOS) method of atmospheric correction was applied to correct the atmospheric effects caused by atmospheric scattering [27]. For this, we used radiometric correction by subtracting the pixel values of dark objects from all pixels in the image scene. The Normalized Difference Vegetation Index (NDVI) [28] was used to calculate the vegetation indices to determine the density of green vegetation cover in the area of interest. The NDVI gives an index of plant greenness or photosynthetic activity. A high value means the area is densely vegetated, while a low value corresponds to less vegetated or barren areas.

**Fig 2:**
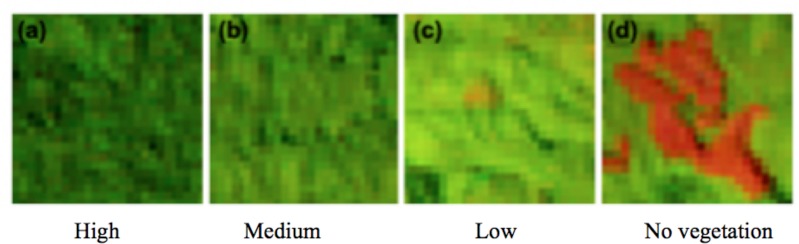
Subset of Landsat image In West Kalimantan showing bands 5, 4 and 3 in RGB combination for vegetation densities

We then applied supervised classification to obtain land cover data [29]. This consisted of recognizing and choosing features on the images and assigning them to a specific category, using the Maximum Likelihood Classification (MLC) method. Dominant land cover classes and densities (high, medium, low and no vegetation) were identified across the study sites (Fig 2).

For the RS desk study land cover classification, polygon patches (30 training samples for each land class category per site) were defined to run the classification method for Papua and West Kalimantan. These training samples represent the same land cover categories (no vegetation, low, medium and high density vegetation and water body) on the image. In Papua because RS detected mangrove, we added this class to the land cover categories for this site only and similarly a category for settlement was added to the West Kalimantan classification.

For Central Java, because we used very high-resolution maps from World Imagery, we were able to derive land features from direct, visual interpretation. Thus, we did not go through the same process of analysis. However we used the same classification as we did for West Kalimantan and Papua, with the additions of the two categories paddy fields and farms.

### Data interpretation

In this article, we look into the complementarity of remote sensing maps used to classify land cover and participatory maps. The villagers first drew the latter maps during community meetings and then together we conducted ground checks. We did not compare the precision of either method because that would need further fieldwork and ground checks would have been needed for validation. However we show that the combination could help further ground checks and validation.

Government agencies generally conduct remote sensing for national-level MRV, using desk-studies, with very little knowledge of the field conditions [4]. In this study, we decided to emulate this approach because we felt this was realistic for many developing countries trying to implement REDD+.

In the first part of the interpretation, we overlaid the land cover maps from both methods (RS and PM) using ArcGIS 10.1® software (ESRI 2011) to identify the overlaps and differences in land cover classification. These overlapping land covers were extracted and stacked in matrices for each research site (Tables 4,5 & 6), with the remotely sensed land cover categories in columns (8 categories in total across sites) and the local people’s land cover categories in rows (39 categories in total across sites). These matrices enabled the team to correlate land cover types from participatory mapping with land cover types identified using remote sensing. This highlights overlaps and areas where local people could complement remote sensing interpretation by adding information about the vegetation types and density. We used maps from Central Java to illustrate the overlaps as they displayed the highest resolution.

**Table 4:** Overlap matrix between remote sensing (RS- vertical) and participatory mapping (PM—horizontal) of land cover classifications for Bagusa and Yoke villages (Mamberamo Raya District, Papua).

PM \ RS	No Vegetation (km^2^)	Mangrove (km^2^)	Low-density vegetation (km^2^)	Moderate density vegetation (km^2^)	High density vegetation (km^2^)
Dense Forest	7.44	0.23	**76.19**	**190.40**	**412.57**
	*4%*	*0*.*%*	***16%***	***33%***	***50%***
Moderately Dense Forest	4.99	0.16	**88.28**	**67.59**	55.76
	*3%*	*0*.*%*	***18%***	***12%***	*7%*
Swamp forest (seasonally flooded)	**32.45**	**18.77**	**264.83**	**299.12**	**283.75**
	***18%***	***13%***	***54%***	***51%***	***35%***
Swampy bush (Permanently flooded)	**97.47**	0.64	32.13	11.35	21.11
	***54%***	*0*.*%*	*7%*	*2%*	*3%*
Fringe Mangrove	0.85	**50.18**	0.99	0.08	3.36
	*0*.*%*	***36%***	*0*.*%*	*0*.*%*	*0*.*%*
Dwarf Mangrove	3.29	**65.49**	12.84	6.88	18.05
	*2%*	***47%***	*3%*	*1%*	*2%*
*Casuarinae sp*. Tree	0.28	0.30	1.60	0.24	2.34
	*0*.*%*	*0*.*%*	*0*.*%*	*0*.*%*	*0*.*%*
Beach	0.09	0.00	0.03	0.00	0.75
	*0*.*%*	*0%*	*0*.*%*	*0%*	*0*.*%*
Marsh	17.15	0.14	2.53	2.60	7.55
	*9%*	*0*.*%*	*1%*	*0*.*%*	*1%*
Water body	16.53	3.84	6.98	6.18	14.65
	*9%*	*3%*	*1%*	*1%*	*2%*
Settlement	0.18	0.04	0.15	0.01	0.33
	*0*.*%*	*0*.*%*	*0*.*%*	*0*.*%*	*0*.*%*
**Total**	**180.72**	**139.79**	**486.55**	**584.44**	**820.22**
	***100%***	***100%***	***100%***	***100%***	***100%***

**Terminology:** *Dense forest–*or tropical rainforest according to Richards and Suryadi [30] is classified as dense by respondents because of tree height, and concentration corresponds, to some extent, to the Indonesian Ministry of Forestry [31] classification of primary dry forest; *moderately dense forest–*classified as moderately dense by respondents, because the stand is more sparse, and trees shorter, due to partial use of this forest for timber extraction and other land uses; *swamp forest*–respondents said this swamp forest is seasonally flooded, especially during the rainy season corresponds, to some extent, to the Indonesian Ministry of Forestry [31] classification of primary swamp forest; *swampy bush–*this land cover is permanently flooded according to respondents, so very little access, but the Indonesian Ministry of Forestry [31] classifies the area as secondary swamp forest; *fringe mangrove–*corresponds to tall mangrove trees bordering canals and rivers; *dwarf mangrove–*smaller mangrove trees (1.5 m) located more inside the mangrove forest, 5 to 20 meters away from the canals; *Casuarinae sp*. *trees—*these trees were planted on the beach in a government program to prevent tidal erosion; *marsh–*land dominated by herbaceous plants on the edge of rivers, lakes and water bodies.

**Note:** Bold numbers are overlaps above 10%.

**Table 5:** Overlap matrix between remote sensing (RS—vertical) and participatory mapping (PM—horizontal) of land cover classifications, for Hulu Pengkadan, Nanga Jemah and Sri Wangi villages (Kapuas Hulu district, West Kalimantan).

PM \ RS	No vegetation (km^2^)	Settlement (km^2^)	Low-density vegetation (km^2^)	Medium density vegetation (km^2^)	High density vegetation (km^2^)
Dense forest	0.06	0.13	0.35	**33.00**	**40.06**
	*2%*	*3%*	*2%*	***17%***	***26%***
Concession logged over area by timber company	0.05	0.25	0.75	**92.36**	**73.86**
	*2%*	*6%*	*5%*	***49%***	***49%***
Past timber company camp	0.01	0.06	0.01	0.44	0.13
	*0*.*%*	*2%*	*0*.*%*	*0*.*%*	*0*.*%*
Secondary forest (30–60 years old)	0.00	0.00	0.02	4.09	4.81
	*0%*	*0%*	*0*.*%*	*2%*	*3%*
Secondary forest < 30 years old	0.00	0.01	0.41	8.45	1.52
	*0%*	*0*.*%*	*3%*	*4%*	*1%*
Mixed rubber and secondary forest (between 30 and 60 years old)	0.02	0.01	0.04	0.50	0.04
	*1%*	*0*.*%*	*0*.*%*	*0*.*%*	*0*.*%*
Smallholder rubber plantation < 3 years old	0.17	0;09	0.10	0.51	0.07
	*6%*	*2%*	*1%*	*0*.*%*	*0*.*%*
Smallholder rubber plantation (3–7 years old)	**0.61**	**0.78**	**4.22**	7.43	0.28
	***22%***	***20%***	***28%***	*4%*	*0*.*%*
Rubber plantation> 7 years old	**0.96**	**1.23**	**6.80**	**20.35**	1.38
	***35%***	***32%***	***45%***	***11%***	*1%*
Waterlogged heath forest	0.00	0.01	0.01	7.59	**21.59**
	*0%*	*0*.*%*	*0*.*%*	*4%*	***14%***
Mixed waterlogged heath forest and dense forest	0.00	0.00	0.00	0.46	4.09
	*0%*	*0%*	*0%*	*0*.*%*	*3%*
Agroforest predominantly durian	0.01	0.01	0.19	0.52	0.05
	*0*.*%*	*0*.*%*	*1%*	*0*.*%*	*0*.*%*
Agroforest predominantly tengkawang	0.00	0.00	0.00	0.05	0.00
	*0%*	*0%*	*0%*	*0*.*%*	*0%*
Cattle pasture	0.02	0.04	0.03	0.05	0.01
	*1%*	*1%*	*0*.*%*	*0*.*%*	*0*.*%*
Upland rice field	**0.47**	**0.88**	**1.53**	3.39	0.18
	***17%***	***23%***	***10%***	*2%*	*0*.*%*
Fallow land	0.18	0.13	0.64	10.16	3.05
	*6%*	*3%*	*4%*	*5%*	*2%*
Paddy (rice) field	0.10	0.00	0.03	0.09	0.00
	*4%*	*0%*	*0*.*%*	*0*.*%*	*0%*
Abandoned artisanal gold mine	0.01	0.02	0.00	0.10	0.03
	*0*.*%*	*0*.*%*	*0%*	*0*.*%*	*0*.*%*
Artisanal gold mine	0.01	0.06	0.02	0.26	0.14
	*0*.*%*	*2%*	*0*.*%*	*0*.*%*	*0*.*%*
Settlement	0.09	0.14	0.02	0.03	0.00
	*3%*	*4%*	*0*.*%*	*0*.*%*	*0%*
**Total**	**2.74**	**3.86**	**15.18**	**189.83**	**151.28**
	***100%***	***100%***	***100%***	***100%***	***100%***

**Terminology:** *Dense forest–*also locally called ‘hutan rimba’ is natural tropical rainforest; *logged over concession area–*regenerated forest that was logged over between 1978 and 1989; *secondary forest–*regenerated tropical rainforest that has been logged either by a timber company or villagers; *agro-forest predominantly durian (Durio sp*.*)*–agroforest with a mix of planted and naturally growing fruit and timber trees, but predominantly durian (fruit tree); *agroforest predominantly tengkawang*–agroforest with a mix of planted and naturally growing fruit and timber trees, but predominantly tengkawang, from the Dipterocarpaceae family belonging to *Shorea* sp. used for the timber and nut. *waterlogged heath forest–*a tropical moist forest found on the island of Borneo also locally called ‘kerapah’.

**Note:** Bold numbers are overlaps above 10%.

**Table 6:** Overlap matrix between remote sensing (RS—vertical) and participatory mapping (PM- horizontal) of land cover classifications, for Lebak and KarangAnyar villages (Wonosobo district, Central Java).

PM \ RS	Settlement (km^2^)	Paddy field (km^2^)	Farm (km^2^)	Low-density vegetation (km^2^)	Medium density vegetation (km^2^)	High density vegetation (km^2^)	Very high density vegetation (km^2^)
Perhutani—(Karang Anyar) pine, with intercropping: albazia and rubber	0.00	0.03	0.00	**0.21**	0.24	0.02	**1.23**
	*0%*	*1%*	*0%*	***19%***	*5%*	*1%*	***89%***
Perhutani 1—(Lebak) pine and albizia with other intercropping	0.00	0.03	0.00	0.00	**0.64**	0.02	0.00
	*0%*	*1%*	*61%*	*0%*	***13%***	*1%*	*0%*
Perhutani 2—(Lebak) pine and mahogany, genitri, albizia, and surian with other intercropping	0.00	0.01	0.00	0.00	0.23	0.10	0.00
	*0%*	*0*.*%*	*0%*	*0%*	*5%*	*7%*	*0%*
Perhutani 3—(Lebak) pine, mahogany, teak and other intercropping	0.00	0.00	0.00	0.06	0.34	0.11	0.00
	*0%*	*0%*	*0%*	*5%*	*7%*	*7%*	*0%*
Agroforest	0.03	**0.47**	0.00	**0.38**	**2.93**	**0.97**	0.09
	*4%*	***17%***	*0%*	***35%***	***59%***	***61%***	*6%*
Chilli Farm	0.00	0.00	**0.00**	0.00	0.00	0.00	0.00
	*0%*	*0%*	***39%***	*0%*	*0%*	*0%*	*0%*
Paddy field	0.00	**2.16**	0.00	**0.44**	**0.55**	**0.37**	0.06
	*0%*	***80%***	*0%*	***40%***	***11%***	***23%***	*4%*
Settlement	**0.78**	0.00	0.00	0.00	0.02	0.00	0.00
	***96%***	*0%*	*0%*	*0%*	*0*.*%*	*0%*	*0%*
**Total**	**0.82**	**2.71**	**0.01**	**1.08**	**4.96**	**1.59**	**1.38**
	***100%***	***100%***	***100%***	***100%***	***100%***	***100%***	***100%***

**Terminology:** *Perum Perhutani or Perusahan hutan Negara Indonesia–*Indonesian state owned timber company; *pine–*dominant species is *Pinus merkussi*; *albazia–*dominant species is *Paraserianthes falcataria*; *rubber–Hevea brasiliensis*; *mahagony*–*Swietenia macrophylla; teak–Tectona grandis; agroforest*–mixed land cover with various crops and fruit trees; *chilli farm–*this particular land cover was distinct because of plastic covering; *paddy field*–irrigated or upland rice fields, annual crops are planted in rotation with rice.

**Note:** Bold numbers are overlaps above 10%.

For the second part of the data interpretation, we made a descriptive analysis by overlaying participatory LU maps with the LC maps derived from supervised classification of the satellite images together with a distance from settlement gradient. Each polygon deliniated from these three proxies was reported in matrixes together with its surface. We used these descriptive (nominal and numerical) data to build Multiple Correspondence Analysis (MCA) using SAS/STAT software, Version (8) 2014 (SAS Institute Inc., Cary, NC, USA The MCA software analyses each line of the matrixes and presents the data set in a two dimensional graph. The X and Y-axes of this graph are called dimensions and are weighted percentages. Graphs allow for quick, visual interpretation using the proximity between each point to indicate the degree of association/correlation–the closer the points, the higher the degree of association. We produced a graph for each province to capture the correlation between land use and land cover in each landscape (the dataset used to build the MCA are available in the supporting information files under S1 Dataset). However, for a matter of concision and clarity, we used only the distance from the settlement as a proxy and chose only to display maps of Sri Wangi Village (West Kalimantan).

Finally, we present an historical map based on local perceptions of past land use and land cover changes. We used one research site, Bagusa Village in Mamberamo Raya District, in which these changes were more visually distinguishable. This map shows historical data gathered during participatory mapping (in FGDs) and some data gathered through 28 key informant interviews on village history and land tenure, four in each village. Four key informants per village were considered sufficient for the requirements of our study. Our key informants were selected because they were knowledgeable, respected members of the community who participated well during the FGDs. During the key informant interviews we used simplified maps to discuss village history, past settlements, displacement and spatial tenure arrangements. All personal information was then encoded in the database during analysis, and only corresponds to a number to maintain the respondents anonymity.

## Results

### 1- Comparing the maps

Land covers, obtained from our land classification analysis of remotely sensed data, provide limited information on land cover types in the absence of ground checks. We found, however, that participatory maps (assembled before RS experts conducted ground checks) provided more detailed information on the different vegetation and/or ecosystem types (Tables 4, 5 and 6). In one area of Papua, for example (Table 4), the local people were able to identify two types of mangrove forest, dwarf and fringe, on the printed satellite maps, while only one type was initially categorised in the remote sensing land classification. During the ground checks they were able to locate these two types of mangrove easily. Similarly, local people differentiated swamp forest from dense forest on dry ground. On the participatory maps, the villagers identified 190 km^2^ of dense forest (the local people consider it dense due to the tree sizes and concentration), 68 km^2^ of moderately dense forest and 299 km^2^ of swamp forest that is seasonally flooded, while the initial classification, based on remote sensing, describes only a moderately dense vegetation covering 584 km^2^.

In Kalimantan the landscape is more a mosaic of natural forest, forest plantations and agricultural land. During participatory mapping, one of the local communities included an area that represented rubber plantations 3 to more than 7 years old (Table 5) in the land class under low-density vegetation. In the village of Nanga Jemah, the local people described their area as different types of land cover: natural dense forest covering 40 km^2^ and natural regeneration from a former timber concession (between 1978 and 1989) covering 74km^2^ while under the land classification, using remote sensing, this area was described as high-density vegetation.

Because of the large areas and important diversity of tree species in Papua and Kalimantan’s natural forests, it was not possible to identify dominant tree species for each land cover from participatory mapping alone. In Central Java, however, because homogeneous planted forests, agroforest and rice cultivation dominate the landscape, the respondents were able to identify dominant species. For example, in Karang Anyar the state owned timber company (Perum Perhutani *Perusahaan Hutan Negara Indonesia*) has forest dominated by pine trees *(Pinus merkussi)* intercropped with albizia (*Paraserianthes falcataria*) and rubber (*Hevea brasiliensis*) for tapping (Table 6), which in the high resolution of satellite imagery of World Imagery, were classified as very high-density vegetation. The villagers also identified other classes of Perhutani forests with mixed stands of pine, albizia, mahogany *(Swietenia sp*.*)*, genitri (*Elaeocarpus ganitrus*) and teak *(Tectona grandis)* have also been identified (Table 6) by villagers.

### Identifying differences

**Fig 3:**
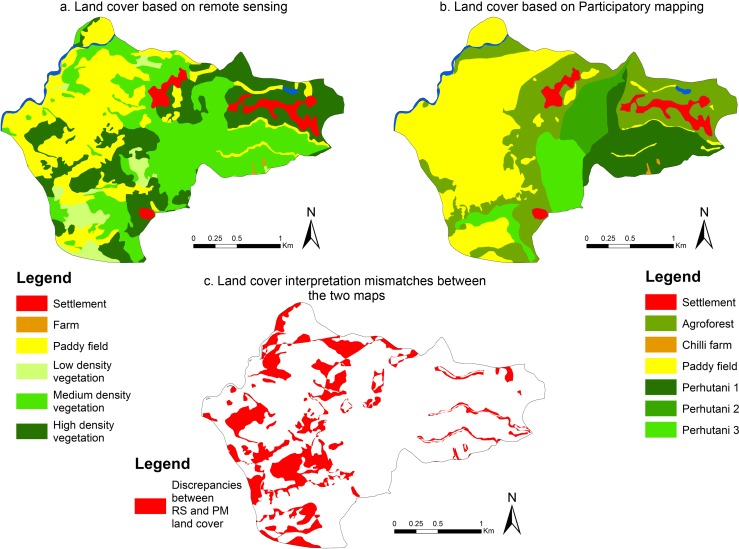
Maps of Lebak (Central Java) illustrating differences in land cover interpretation between RS and PM

The maps of Central Java (Fig 3) give an idea of the possible differences and overlaps between participatory mapping methods and remote sensing in delineating land cover. The Central Java study sites cover smaller areas (about 6 to 7 km^2^), compared to our other sites (between 27 and 1400 km^2^), and present the highest resolution of satellite images (1 m) allowing a better interpretation of satellite data. From World Imagery we were able to see small features such as individual paddy fields, rooftops, roads, paths and farming practises (e.g. chilli farms) from the high-resolution satellite images. These results, however, are not necessarily in agreement with maps from participatory processes though we used the same World Imagery maps as base maps for both methods (Fig 3). We overlaid the two maps and highlighted differences in land cover (Fig 3C) reported in Table 7.

**Table 7:** Percentage of contradicting overlaps in representation of the land cover by both methods (Central Java).

LC from Participatory mapping	LC from Remote sensing	Differences (%)
**Lebak**		
Agroforest	Paddy field	13.95%
Chilli pepper farm	Paddy field and medium vegetation density	62.73%
Paddy field	High to low-density vegetation	35.59%
Perhutani 1	Paddy field and farm	5.30%
Perhutani 2	Paddy field	2.58%
Perhutani 3	Paddy field	0.48%
**KarangAnyar**		
Agroforest	Paddy field, river and settlement	8.23%
Paddy field	River, settlement, low to very high-density vegetation	46.84%
Perhutani	Paddy field and settlement	2.07%
Settlement	Paddy field, medium density vegetation	3.07%
River	Paddy field, medium density vegetation	6.85%

For instance, local people described agroforest in areas where land classification analysis identified paddy fields. These differences covered 14% of the paddy fields. There was also a 36% overlap of paddy fields (PM) and low to high-density vegetation (RS).

### 2- Linking local land use to land cover

**Fig 4:**
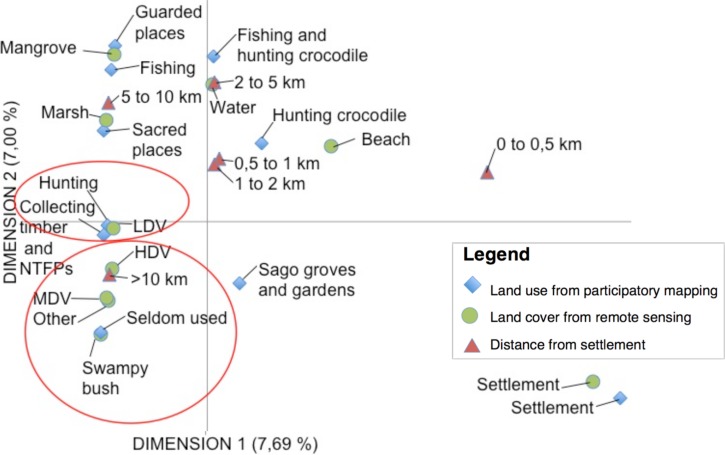
Multiple Correspondence Analysis (MCA) for Papua. (LDV: Low-density vegetation; MDV: Medium density vegetation; HDV: High density vegetation; VHDV: Very high density vegetation; NTFP: Non-timber forest product)

We used Multiple Correspondence Analysis (MCA) to draw clear correlations between land cover (vegetation density) from land classification maps and local land use, which correlated to the dominant land use found in each land cover. In Papua (Fig 4), we found strong correlations between hunting, collecting timber and NTFPs (land use) with low-density vegetation. This was also verified using data from our key informant interviews. The forests where 43% of the people interviewed go for these purposes, within a 2 to 10 km radius of the village settlement, were covered in low-density vegetation. On the other hand, in swampy bush, an area permanently flooded according to villagers, we found a correlation with seldom-used areas–minimal hunting, and gathering of timber and NTFPs. Other correlations could be made from this MCA, especially using distance from a settlement as a proxy. For example, high and medium-density vegetation are found farther than 10 km from the village settlement, which indicates a decrease in land use and an increase in vegetation density the further we are from a village settlement.

**Fig 5:**
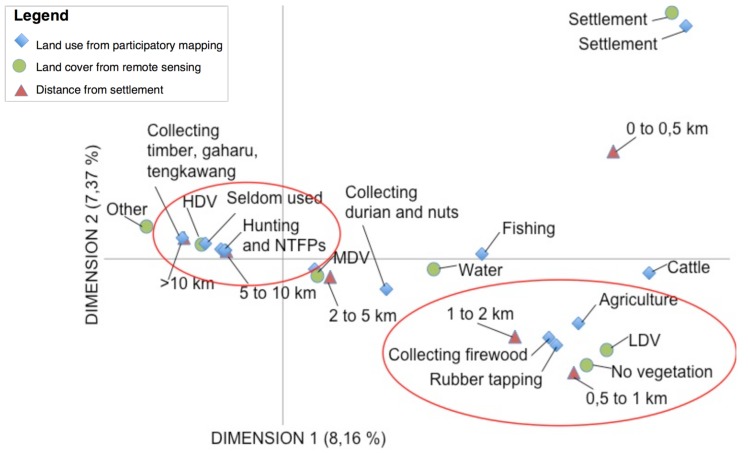
Multiple Correspondence Analysis (MCA) for West Kalimantan. (LDV: Low-density vegetation; MDV: Medium density vegetation; HDV: High density vegetation; VHDV: Very high density vegetation; NTFP: Non-timber forest product)

In West Kalimantan (Fig 5), the MCA allowed us to correlate non-vegetated and low-density vegetation classes from land classification. These were derived from supervised classification, with other land use such as agriculture, rubber tapping and collecting firewood, from participatory maps, within a 0.5 to 1 km radius of the village settlements.

**Fig 6:**
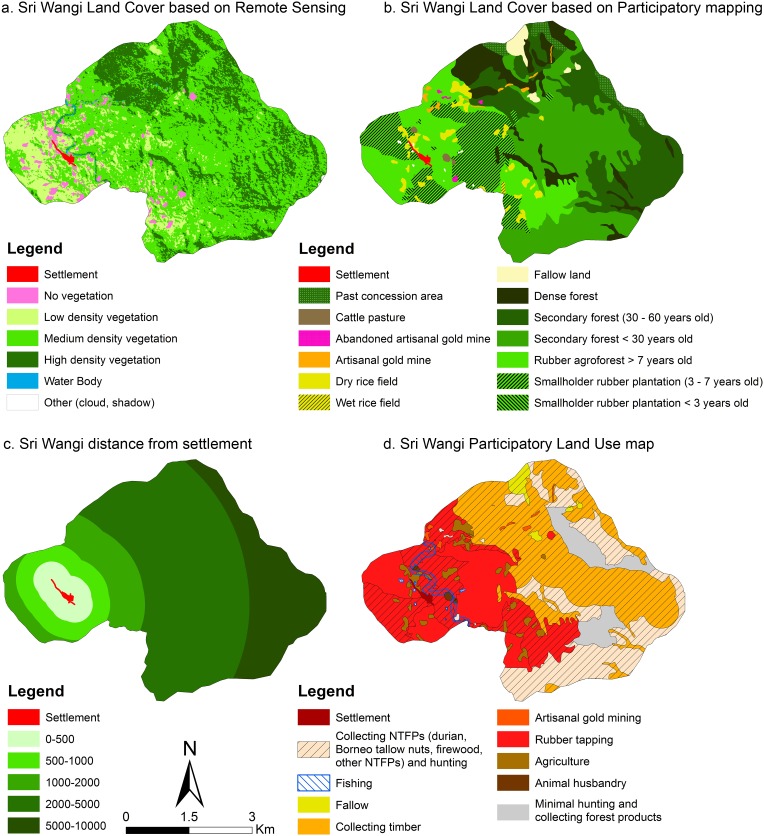
Maps of Sri Wangi (Kalimantan) showing a correlation between land cover (LC) using remote sensing (map a.), participatory mapping (map b.), distance from settlement (map c.) and participatory land use (map d.)

If we look at Fig 6, we can see that Sri Wangi has agriculture and smallholder rubber plantations (between 3 and more than 7 years old) in close proximity to the village settlement (0 to 2 km). Intensity of land use correlates negatively with distance from settlements. For example, within a 2 to 5 km radius, there is a strong correlation with medium-density vegetation, hunting and NTFP collection. We found correlations between high-density vegetation and seldom-used areas, collecting timber, agarwood and tengkawang beyond 5 km distance from settlements. The area the land classification map, from supervised classification, identifies as no vegetation (Fig 6 map a.), might be a result of crop rotation. The land classification map may have picked up bare land cleared for upland rice cultivation, traditionally cultivated for 2 years before planting rubber. And low-density vegetation might also be associated with young plantations.

**Fig 7:**
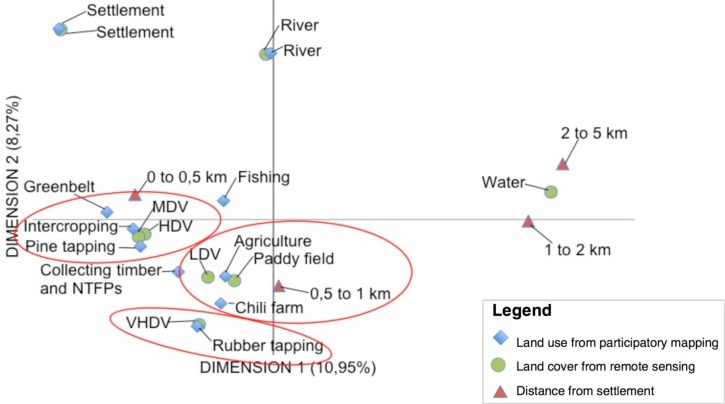
Multiple Correspondence Analysis (MCA) for Central Java. (LDV: Low-density vegetation; MDV: Medium density vegetation; HDV: High density vegetation; VHDV: Very high density vegetation; NTFP: Non-timber forest product)

In Central Java, MCA (Fig 7) shows a high correlation between paddy fields and low-density vegetation, from land classification of the high-resolution satellite imagery, with agriculture and chilli farms on participatory maps. In closer proximity to village settlements (0 to 0.5 km) high and medium vegetation density were found associated with villagers’ activities such as intercropping, pine tapping and collecting NTFPs that are usually conducted in Perhutani forests. Also very high-density vegetation in Karang Anyar Village is where the villagers intercrop Perhutani forest (pine trees) with rubber (Table 6).

### 3- Mapping historical change in customary territory

During participatory mapping, from focus group discussions and key informant interviews we collected information on village history including displacement of the village settlement, former garden locations, hunting and gathering places, and a sense of spatial tenure arrangement.

**Fig 8:**
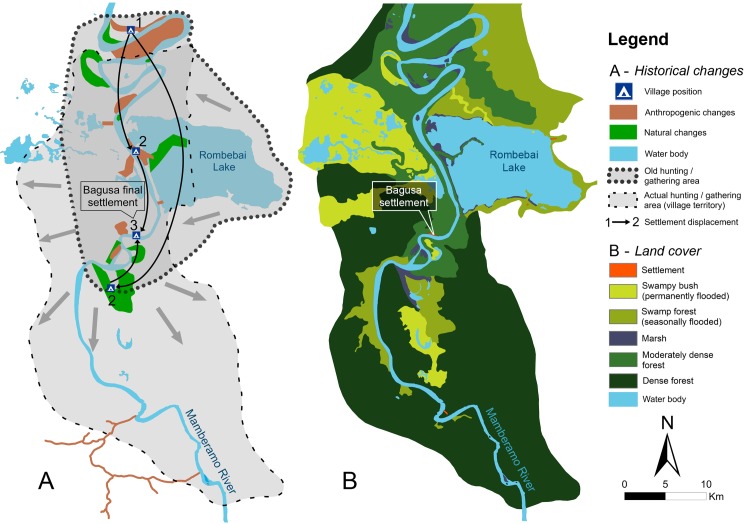
Map of Bagusa village, Papua, displays settlement displacement, change in hunting area, natural and anthropogenic LU and LC changes

In Papua, areas where people obtain resources important for their livelihoods are usually used to mark their territory. Village history, past settlements and old gardens (e.g. ancestral sago groves) also play an important role in marking the village territory. These territories could be exclusive as well as shared with neighbouring villages. In the Bagusa territory, for example, people from neighbouring villages that share a common history or ancestor(s) are granted access to hunt and collect food and other forest products for subsistence. Fig 8 shows the former hunting and gathering areas that have expanded further south following the village’s displacement to higher ground. The expansion of the territory is also due to population growth. These hunting and gathering areas (grey with a dashed border on the map, see Fig 8) are where villagers from Bagusa gather timber, NTFPs and hunt, and the area in which they might have the biggest impact.

Fig 8 shows the changes attributed to either natural or anthropogenic causes. This information was gathered during the mapping of past LC and LU. Natural changes are mostly due to river erosion or seasonal flooding. While anthropogenic changes are due to human activities that shape the forest such as slash and burn practises for opening gardens and digging canals to access lakes. This gives a better understanding of how local people are shaping their territory, which could be useful when analysing LC change.

## Discussion

Dharmadi Hawthorne and Boissière [32] highlight current gaps in research on participatory MRV. Research on the possible role of local communities in carbon estimations using remote sensing and ground checks, is often missing. Here, we suggest that involving local communities in REDD+ MRV, using participatory mapping as a first check, will provide valuable social data such as local land use, tenure arrangements, etc., information essential for land classification (derived from supervised classification) of satellite images. We expect that community participation in ground truthing and measurements will increase the information on complex changes in forest cover.

Community participation is especially relevant where countries use RS to monitor REDD+ implementation. For instance, in Papua, land classification categorised an area as ‘bare land’ covering 180km^2^, however, the villagers identified this area as mostly swampy bush (98km^2^) and swamp forest (33km^2^). Getting the right information is especially important in carbon estimations. Using the tier 2 approach [5] if we classify this area of ‘bare land’ as grassland for a higher carbon estimate, the above ground carbon stock per ha would be between 2 to 4 Mg.ha^-1^ [33], whereas swampy bush would be between 18 and 35 Mg.ha^-1^ [33] and swamp forest between 90 and 200 Mg.ha^-1^ [33]. In another example from Papua, land cover classification categorised an area as one type of mangrove when the local people identified it as a mix of dwarf mangrove (66 km^2^) and fringe mangrove (50 km^2^) [34]. This would make a significant difference in terms of carbon stock if not differentiated. Dwarf mangroves store at least 8 Mg.ha^-1^, while fringe mangroves store more than 500 Mg.ha^-1^ [35]. In another example from West Kalimantan, villagers identified an area as secondary or regenerated forest from a timber concession operating between 1978 and 1989, while the initial land classification without official data categorised the same area as natural forest, which would effect carbon estimations [36]. The preceding carbon stock estimates are based on the literature and do not take into consideration the validation of RS analysis through ground checks nor measurements of the carbon stock in each land type.

GOFC-GOLD suggests the use of time series to estimate carbon emissions [5]. Participatory mapping could follow these regular intervals to monitor land use and land cover at the same time. However, this kind of information is limited to locations where people live and have knowledge of. However, areas far from settlements need to be validated through more conventional ways and because local people have a better knowledge of these remote places, they may want to conduct ground checks and/or measurements. Local capacity could be increased to estimate carbon for REDD+ national initiatives [37–39]. Chhatre and Agrawal [40] noted that this kind of monitoring creates a sense of local ownership and value of forests, crucial to their long-term acceptance and to the sustainability of PMRV. Also studies in remote areas have highlighted the role of local people and their autonomous monitoring in deterring illegal logging, encroachment and poaching [41] often with no acknowledgment. Incorporating local monitoring into MRV could help detect early forest degradation and deforestation and improve cooperation between local people and authorities to stimulate local action for rapid forest management interventions [42].

GOFC-GOLD [5] in its guidelines for REDD+ carbon measurements, suggests that sampling efficiency can be improved through spatial stratification using a known proxy (e.g. deforestation hotspots). Overlaying both maps would help reduce sample size and stratify ground check areas to focus on LC overlaps and differences, such as those presented for Central Java. Zhang et al. [43] integrated participatory processes with GIS multiple criteria to determine suitable zones for conservation. Using the same principle, the overlay of information from land cover, local land use, distance from settlement, access routes, etc., could help to determine suitable areas for stratified measurement plots and increase confidence in carbon stock estimations, while saving time and money.

The UNFCCC negotiations [16, 44] have encouraged developing countries to identify land use, land use change and forestry activities, in particular those that are linked to the drivers of deforestation and forest degradation [45]. While deforestation seems easy to detect using remote sensing [2], forest degradation generally results in small changes in canopy cover (e.g. small scale deforestation), which RS cannot detect, hiding the real extent of deforestation and forest degradation [46].

Correlating local people’s land use with patterns in forest cover can help us to understand variations in the vegetation dynamics. For example, results from the Multiple Correspondence Analysis (MCA) in West Kalimantan sites highlight the link between rubber tapping and low-density vegetation or no vegetation. Other variables should also be selected as proxies such as distance from settlements or travel axes (e.g. roads, rivers) etc. We found that vegetation density is also positively correlated with the distance from the settlements. While this may be an obvious hypothesis, the maps and the MCA confirm and give a clear picture of how the villagers extend their influence over forest vegetation. Although validating such an assumption seems easy, validating different proxy analyses may prove more difficult to interpret. Thus, we need to be careful not to generalize or extrapolate correlations as environmental and social conditions may differ from site to site [47] as well as in time.

One limitation with time series is the availability of reliable satellite imagery for a given period. For our research we had to use different dates (Table 3) for the different sites. We wanted to work in similar conditions as the Indonesian national forest inventories, using the same free source of satellite imagery. We also selected satellite images that had the lowest cloud coverage, which was particularly difficult to obtain for Papua. The multi-temporal data are also affected by seasonal factors [47]. This must be taken into account when designing MRV. Because forest change can happen rapidly and crop rotation in shifting cultivation changes every few years, monitoring forest change may require of more regular and closer intervals. In West Kalimantan a plantation of young rubber (2 years old) and paddy fields were not picked up due to time differences. This underlines the need to simultaneously conduct participatory mapping and remote sensing analysis in a relevant and timely manner, necessitating more regular contact between the government and local communities, which could create stronger links between the two. During our fieldwork, we were the first to develop a map of these community territories and acknowledge customary claims to forest and land.

Drawing the historical context (past LU, LC and drivers of change) of the villages (Fig 8) on the maps revealed the impacts of previous settlements and plantations and shifting cultivation have had on forests. This can be correlated with land cover change (LCC) from land classification maps derived from supervised classification. However, there are risks in using PM that the villagers may have forgotten exactly where and when events took place [47]. The absence of documentation and maps (e.g. community forest boundaries, land claims, forest management and land use planning) also played an important role in that uncertainty [48]. Such documentation exists and is exhaustive in Central Java, but is absent in Papua and incomplete in West Kalimantan, highlighting the need for participatory mapping of site specific, local history and activities.

Participatory maps can also help identify potential conflict in land use [49]. This is especially true in the case of Indonesia where statutory tenure is often disconnected from the local *defacto* (indigenous) tenure and therefore a source of conflict [50]. Discussion between the government and the local community on the recognition of indigenous rights and tenure claims is urgently needed, not only in the context of REDD+, but in other contexts as well.

The Indonesian Geospatial Information Act of 2011 encourages customary mapping through land registration; and individual, group or corporate participation in the identification of land parcel boundaries as part of a national program [51], which will help measure the impact of REDD+ in the future. Furthermore, social safeguards for REDD+ require participation and respect for local and indigenous community rights [52] including the recognition of customary rules [53]. The keen interest of local communities in drawing the maps is a good indicator of their willingness to debate these issues. They also believed that the maps could help them succeed in their territorial claims and expel outsiders [54].

Participatory monitoring of land use changes, combined with RS, could quickly verify of REDD+ targets, assess the effectiveness of REDD+ interventions and provide local communities with incentivised alternative livelihoods [55]. Regular RS and PM of land use and its implications for land cover together with livelihood and social data (e.g. tenure, source of income for local communities) will help monitor changes in local livelihood under REDD+ activities and should be analysed when distributing benefits.

Drawing maps can help local people to visualize the space and scale of their daily activities and bring different issues to the table for discussion. For example, Gaillard et al. [56] used participatory mapping in Nepal to encourage multi-caste collaboration in climate change adaptation and disaster risk reduction. But the representativeness of participatory mapping is under debate. Gaillard et al. [56] call for caution on the process of validation as experience demonstrates that if not conducted carefully, participatory mapping can represent a small group of the respondents’ perceptions to the detriment of the rest of the community. Plus, formalising indigenous participation is not enough to break through existing power structures that can prevent marginalized stakeholders from defending their interests [57]. Any participatory mapping activities must have ethical considerations ensuring that mapping outputs are fully understood by all concerned, and traditional knowledge and its ownership are respected and protected [58, 59].

## Conclusion

The impact of land use on forest conditions is site specific and cannot be separated from the local context (e.g. economic, demographic pressure, governance) hence the need for local monitoring. In this way local people will be able to play a key role in the measurement of carbon sequestration and provide important long-term information on forest dynamics. However, the connection between REDD+ MRV and local communities, which will provide information on the efficiency of REDD+ and its impact on local livelihoods, is perhaps the most important aspect of such a partnership.

As valued stakeholders, local communities would be more likely to join a partnership in monitoring and protecting sensitive hotspots, particularly if their motivations to participate were addressed. Participatory mapping is only one of the many tools to capture local knowledge and local points of view, but it is not enough to secure local participation in REDD+ MRV. Local communities need to see a direct benefit, be it financial or other, for contributing to the pool of information needed for REDD+. There is not one single solution for all situations. Participatory mapping must be adapted to the knowledge of different ecosystems, meet the interests of local communities in using them and be useful to other relevant stakeholders. The potential of participatory maps should not be underestimated especially in association with Remote Sensing, as together they could play a key role in initiating discussion among all stakeholders on land use arrangements and tenure issues, paramount to the success and sustainability of REDD+.

## Supporting Information

S1 Dataset Data Set for MCA. This file includes the different numerical and nominal data used to build the Multiple Correspondence Analysis (MCA) graphs displayed in Fig 3. The first sheets in this file represent the raw data. The final three sheets that include the graphs, are X and Y coordinates generated by SAS/STAT software, Version [8] 2014 (SAS Institute Inc., Cary, NC, USA).
(XLSX)
